# Angiotensin II promotes the anticoagulant effects of rivaroxaban via angiotensin type 2 receptor signaling in mice

**DOI:** 10.1038/s41598-017-00473-5

**Published:** 2017-03-23

**Authors:** Dan Yang, Junjie Shao, Ruifeng Hu, Haimei Chen, Ping Xie, Chang Liu

**Affiliations:** 1 Key Laboratory of Bioactive Substances and Resources Utilization of Chinese Herbal Medicine from Ministry of Education, Institute of Medicinal Plant Development, Chinese Academy of Medical Sciences and Peking Union Medical College, Beijing, 100193 P.R. China; 2 Department of Pathology, Shanghai KingMed Diagnostics, Shanghai, 201321 P.R. China

## Abstract

Rivaroxaban is an oral direct factor Xa inhibitor approved for the treatment of stroke and systemic thromboembolism in patients with non-valvular atrial fibrillation. Despite its efficacy, rivaroxaban therapy results in adverse effects and complications, such as bleeding. Angiotensin II (AngII) is implicated in many cardiovascular conditions, such as hypertension and heart failure. In this study, we investigate whether AngII influences anticoagulant effects of rivaroxaban by using an experimental mouse model with type 2 diabetes mellitus and advanced glycation end product **(**AGE)-exposed human umbilical vein endothelial cells (HUVECs). We found that AngII promoted the anticoagulant effects of rivaroxaban in KKAy mice. The combination of rivaroxaban and AngII enhanced *in vivo* tissue factor pathway inhibitor (TFPI) activity and induced TFPI expression and activity in AGE-exposed HUVECs. Angiotensin type 2 receptor (AT2R) and Mas antagonists attenuated the AngII-enhanced anticoagulant action of rivaroxaban *in vivo*, and abolished the increased endothelial TFPI expression and activity. However, angiotensin type 1 receptor (AT1R) antagonist exerted no effects. Additionally, combination of rivaroxaban and AngII induced aortic AT2R and Mas expression. Our data suggest that the anticoagulant effects of rivaroxaban are promoted by AngII via AT2R and Mas signaling. These findings are significant for the clinical administration of rivaroxaban.

## Introduction

Coagulation is a process in which blood changes from a liquid to a gel and is the main cause of clot formation. It could result in hemostasis, the cessation of blood loss from a damaged vessel, followed by vessel repair. Malfunctions of the coagulant system are implicated in various pathological states, such as hypercoagulability, thrombosis, and inflammation. Furthermore, chronic diseases are frequently accompanied by hypercoagulable state, which collude closely with inflammatory processes^1^. Coagulation is initiated when blood is exposed to cells expressing the tissue factor (TF). TF is a physiological constituent under the endothelial lining in blood vessels; it forms a protective hemostatic layer that limits bleeding after vessel injury. TF can bind and activate factor VII (FVII), lead to the activation of factor X (FX) and ultimately blood clot formation. TF-initiated coagulation is under the control of a TF pathway inhibitor (TFPI), a Kunitz-type serine protease inhibitor predominantly produced by endothelial cells. TFPI is a single-chain polypeptide and forms a stable complex with TF and activated FVII (FVIIa) to inhibit the function of activated FX (FXa) reversibly. When FXa is inhibited, the TFPI/FXa complex can subsequently inhibit the function of TF/FVIIa complex^2^.

Rivaroxaban is the first oral direct FXa inhibitor approved for the prevention and treatment of venous thromboembolism or stroke prophylaxis in atrial fibrillation (AF). Rivaroxaban exerts its anticoagulant effects by forming two hydrogen bonds with the amino acid Gly219 of FXa. However, adverse effects and complications, such as gastrointestinal bleeding, have been associated with rivaroxaban therapy^3^, and the incidence and risk factors for bleeding vulnerability have not been well established in patients who take rivaroxaban.

The renin–angiotensin system (RAS) plays a crucial role in the physiological regulation of blood pressure, sodium and fluid balance, and vascular tone. The octapeptide angiotensin II (AngII) is the primary mediator of this system and has been implicated in diverse human diseases, such as hypertension, congestive heart failure, myocardial infarction, aneurysm, stroke, and diabetes. A key question is how AngII performs many pleiotropic functions under different conditions. One of the explanations concerns its different receptor types.

AngII can bind to at least two types of high-affinity receptors, namely, angiotensin type 1 receptor (AT1R) and angiotensin type 2 receptor (AT2R). Primarily, AT1R is expressed in adults, whereas AT2R is expressed in fetuses. However, AT2R can reoccur in adults ﻿under pathophysiologic conditions, such as cardiovascular injury and remodeling^4, 5^. Most of the known pathophysiologic effects of AngII are mediated by AT1R, including vasoconstriction, increased blood pressure, inflammation, fibrosis induced by chronic inflammation, increased oxidative stress, and aldosterone production. By contrast, the biological effects of AT2R are largely unknown. A few studies have indicated that AT2R activation counteracts most of the effects of AT1R by suppressing cell proliferation and differentiation, promoting vasodilation, and repressing inflammation and oxidative stress^4, 5^. Recently, AngII was implicated in coagulation abnormality and thrombogenesis in clinical trials and animal studies^6, 7^. Elevated AngII levels in animals, elicited by either genetic alteration or chronic AngII infusion, were accompanied by increased TF expression and plasma plasminogen activator inhibitor-1 (PAI-1) levels^7^.

Previously, we observed that the combination of rivaroxaban and AngII increased the occurrence of gastrointestinal bleeding in KKAy mice. In the current study, we carried out in depth investigations to understand the molecular mechanisms underlying this observation.

## Results

### AngII promoted the anticoagulant effects of rivaroxaban in KKAy mice

**Figure 1:**
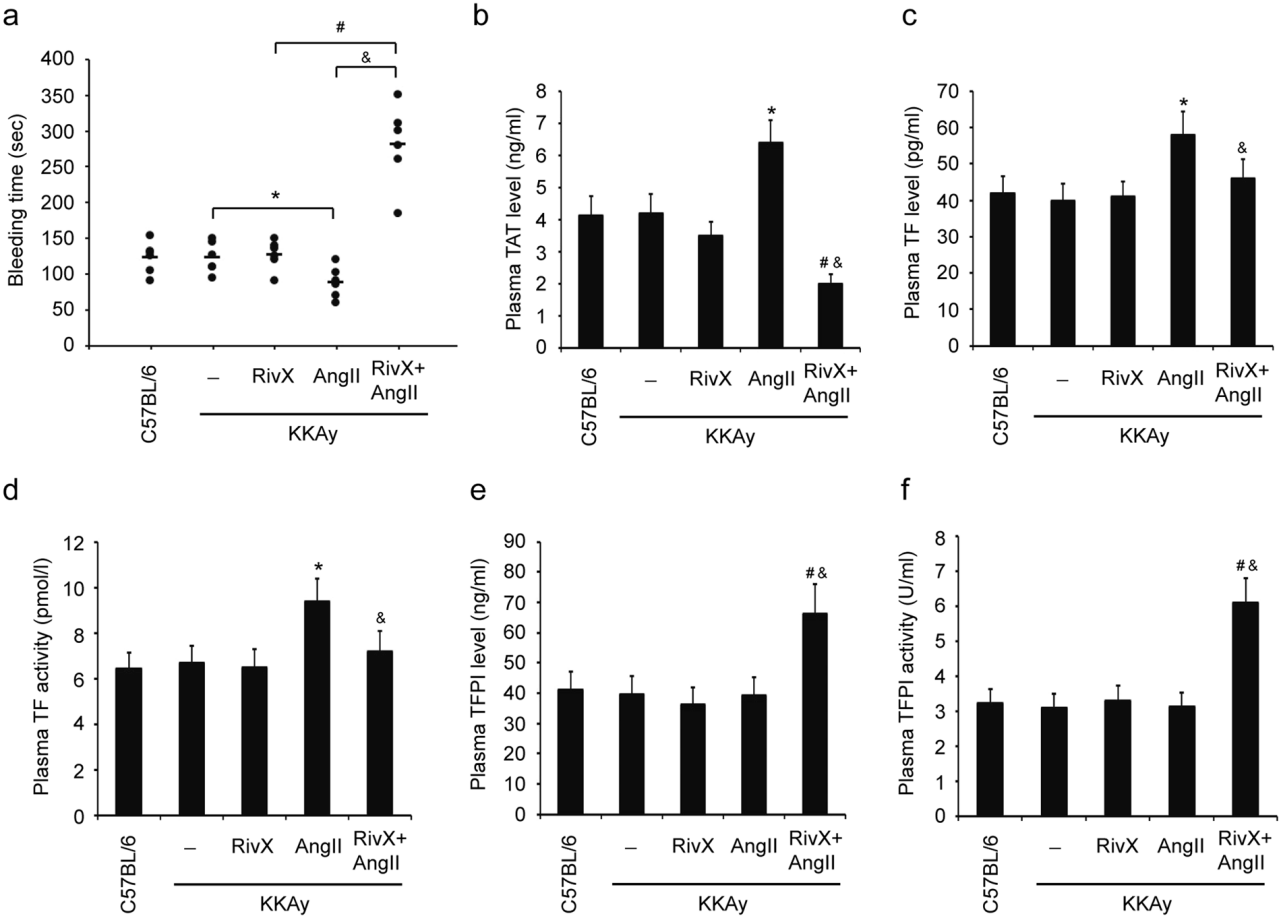
AngII promoted the anticoagulant effects of rivaroxaban in KKAy mice. Mice were administered orally with rivaroxaban (RivX, 5 mg/kg body weight/day), or infused with AngII (1,500 ng/kg/min), or co-treated with RivX and AngII for 2 weeks. (**a**) Under isoflurane anesthesis, bleeding time was assessed. Blood was collected by cardiac puncture to determine the plasma levels of thrombin-antithrombin (TAT) complex (**b**), tissue factor (TF) (**c**), and TF pathway inhibitor (TFPI) (**d**), as well as the plasma activities of TF (**e**) and TFPI (**f**). The bars show the mean ± SEM (n = 6 to 8). ^*^ *P* < 0.05, compared with the KKAy blank group; ^#^ *P* < 0.05, compared with the RivX treatment group; ^&^ *P* < 0.05, compared with the AngII treatment group.

We examined the anticoagulant effects of rivaroxaban by using an experimental mouse model with type 2 diabetes mellitus. Firstly, bleeding time in KKAy mice was comparable to that in C57BL/6 control mice (Fig. 1a). After oral administration of rivaroxaban for 2 weeks, bleeding time in KKAy mice was prolonged slightly without statistical significance. In agreement with the procoagulation function of AngII documented previously, KKAy mice infused with AngII showed a shorter period of bleeding time (Fig. 1a). Surprisingly, combination of rivaroxaban and AngII significantly increased the bleeding time compared with rivaroxaban alone or AngII alone group (Fig. 1a). These changes coincided with reductions in the plasma levels of TAT (Fig. 1b). However, no parallel changes in plasma TF levels (Fig. 1c) and activities (Fig. 1d) were detected. Interestingly, in contrast to treatment of rivaroxaban alone or AngII alone, the combination of both significantly increased the expression levels (Fig. 1e) and activities (Fig. 1f) of TFPI in plasma. Additionally, we did not detect obvious changes in the protein levels of anticoagulation thrombomodulin (TM) (Supplementary Figure 1a and b) and procoagulant von Willebrand factor (vWF) (Supplementary Figure 1c). These results suggested that AngII promoted the *in vivo* anticoagulant effects of rivaroxaban, and TFPI might play a role in this process.

### Rivaroxaban did not suppress procoagulation cytokine levels in the presence of AngII

**Figure 2:**
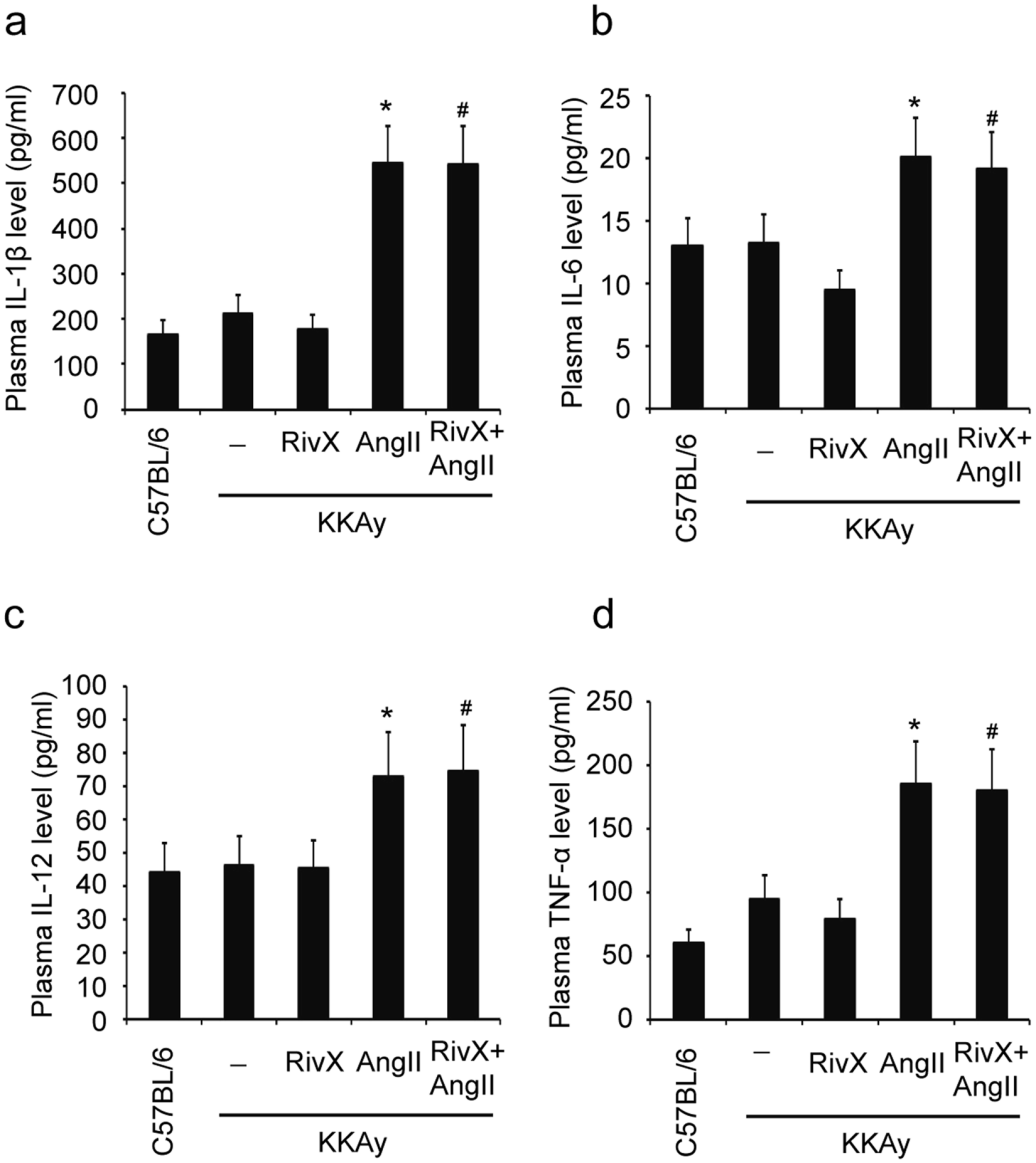
Rivaroxaban did not suppress procoagulation cytokine levels in the presence of AngII. Mice were administered orally with rivaroxaban (RivX, 5 mg/kg body weight/day), or infused with AngII (1,500 ng/kg/min), or co-treated with RivX and AngII for 2 weeks. Blood was collected by cardiac puncture to determine the plasma levels of IL-1β (**a**), IL-6 (**b**), IL-12 (**c**), and TNF-α (**d**) using ELISA. The bars show the mean ± SEM (n = 6). ^*^ *P* < 0.05, compared with the KKAy blank group; ^#^ *P* < 0.05, compared with the AngII treatment group.

Inflammation activates coagulation under various pathological conditions. Proinflammatory cytokines, including IL-1β, IL-6, IL-12, and TNF-α, function as stimuli for a state of hypercoagulability^1^. Accordingly, we examined plasma proinflammatory cytokine levels after treatment of mice with rivaroxaban plus AngII. As shown, compared with administration of rivaroxaban alone, addition of AngII led to increased plasma levels of IL-1β (Fig. 2a), IL-6 (Fig. 2b), IL-12 (Fig. 2c), and TNF-α (Fig. 2d). Given that IL-1β, IL-6, IL-12, and TNF-α are stimuli for a hypercoagulable status, the enhanced anticoagulant effects of rivaroxaban in the presence of AngII were less likely caused by the actions of proinflammatory cytokines.

### The AT2R and Mas antagonists attenuated AngII-enhanced anticoagulant effects of rivaroxaban

**Figure 3:**
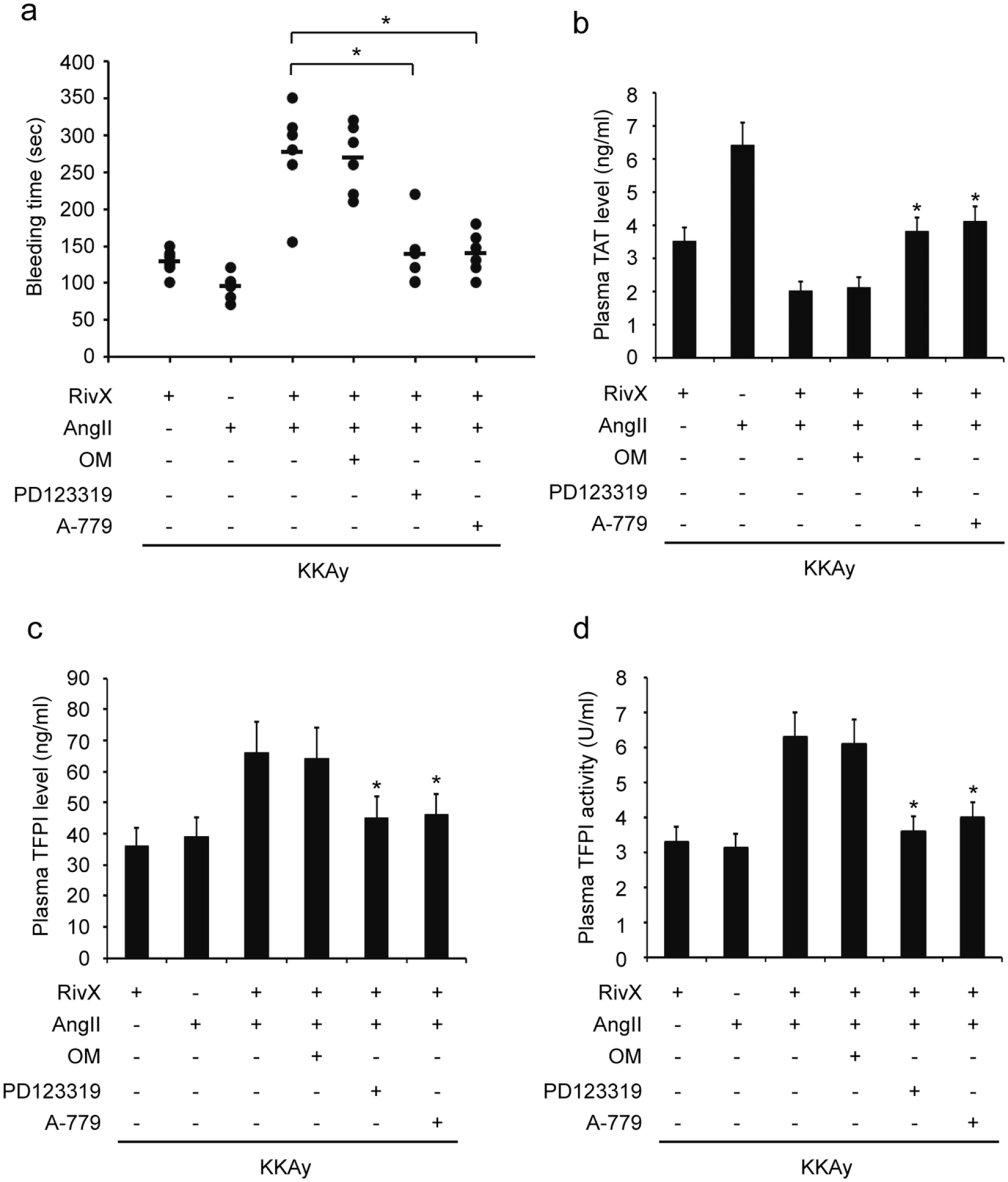
AT2R and Mas antagonist attenuated the AngII-enhanced anticoagulant effects of rivaroxaban. Mice were administered with rivaroxaban (RivX, 5 mg/kg body weight/day) or AngII (1,500 ng/kg/min) in the presence of AT1R antagonist olmesartan medoxomil (OM, 0.5 mg/kg/day), or AT2R antagonist PD123319 (3 mg/kg/day), or Mas antagonist A-779 (2 mg/kg/day) for 2 weeks. (**a**) Under isoflurane anesthesis, bleeding time was assessed. Blood was collected by cardiac puncture to determine the plasma levels of thrombin-antithrombin (TAT) complex (**b**) and TF pathway inhibitor (TFPI) (**c**), as well as the plasma activities of TFPI (**d**). The bars show the mean ± SEM (n = 5–6). ^*^ *P* < 0.05 compared with the parallel RivX and AngII co-treated group.

Having shown the enhancement of the anticoagulant effects of rivaroxaban by AngII, we examined whether AngII receptors were implicated in these pathological processes. The increase in bleeding time by co-administration of rivaroxaban and AngII was largely abolished by the AT2R antagonist PD123319 without obvious effects caused by the AT1R antagonist olmesartan medoxomil (Fig. 3a). In contrast to combination of rivaroxaban and AngII, the addition of PD123319 increased the plasma TAT level; the addition of olmesartan medoxomil exerted no such effects (Fig. 3b). On the other hand, the plasma levels and activities of TFPI were reversed significantly by PD123319, and olmesartan medoxomil exerted no effect (Fig. 3c and d). The biologic actions of AT2R and Mas receptor and their signaling mechanisms display striking resemblances. Therefore, we inhibited Mas to detected anticoagulant effects of rivaroxaban in the presence of AngII. In agreement with these, Mas receptor antagonist A-779 showed similar effects with that of PD123319 (Fig. 3). Moreover, plasma TF levels and activities were significantly inhibited by olmesartan medoxomil, and promoted by PD123319 and A-779 (Supplementary Figure 2a and b). Additionally, olmesartan medoxomil, PD123319, or A-779 itself had no effects on the bleeding time (data not shown). Overall, it appeared that AT2R and Mas receptor signaling contributed to the enhanced anticoagulant effects of rivaroxaban in the presence of AngII.

### Rivaroxaban induced AT2R and Mas expression in the presence of AngII

**Figure 4:**
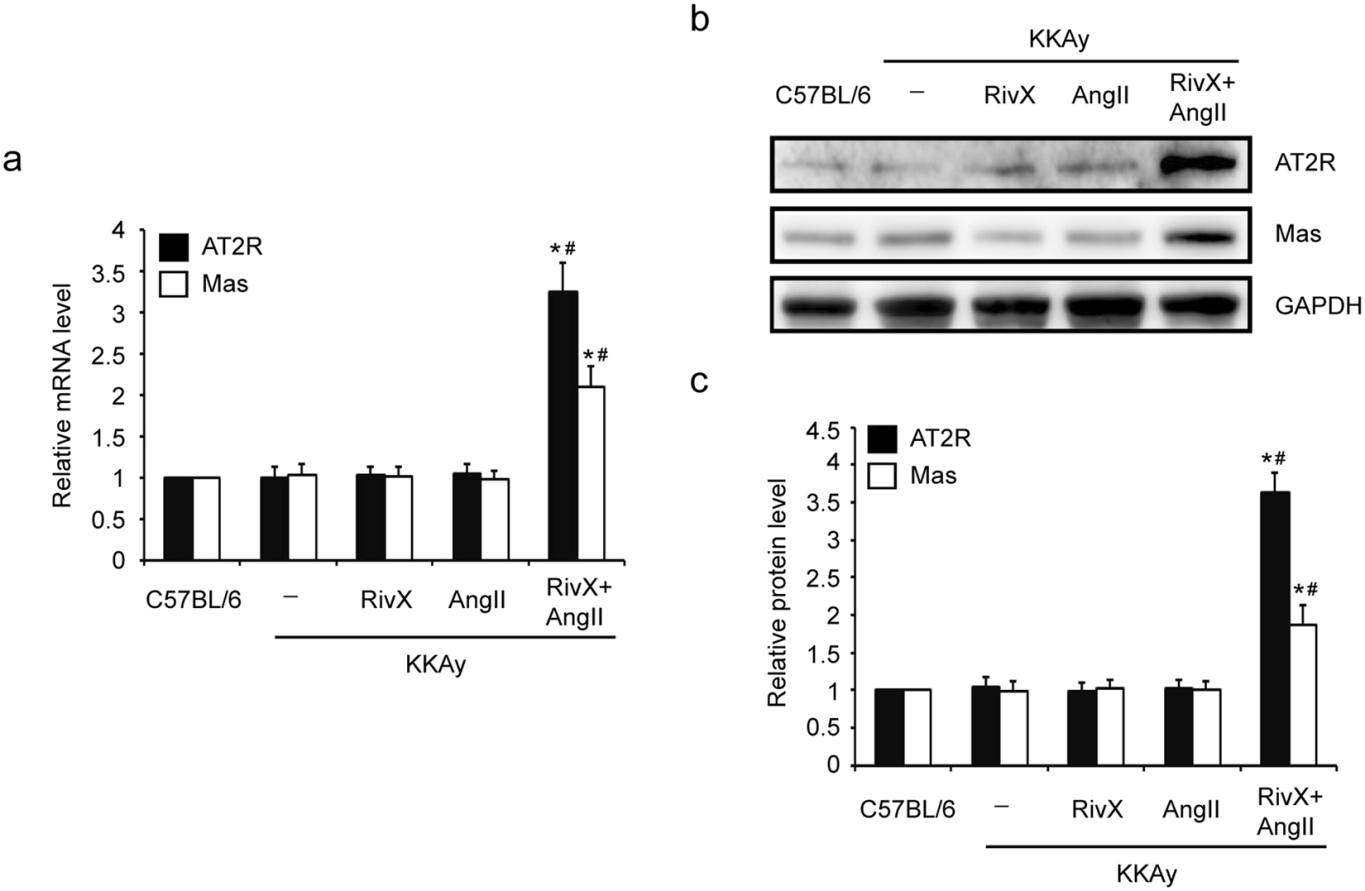
Combination of rivaroxaban and AngII up-regulated the expression levels of AT2R and Mas. Mice were administered orally with rivaroxaban (RivX, 5 mg/kg body weight/day) or infused with AngII (1,500 ng/kg/min) or co-treated with RivX and AngII for 2 weeks. Under isoflurane anesthesis, the aortae were harvested. (**a**) The relative mRNA expression levels of AT2R and Mas were detected. The bars show the mean ± SEM (n = 5). ^*^ *P* < 0.05 compared with the group treated with RivX alone, and ^#^ *P* < 0.05 compared with the group treated with AngII alone; (**b**) The protein expression levels of AT2R, Mas, and GAPDH were detected using Western blot analysis. Cropped Images for representative blots were shown. Images for the full-length blots can be found in the supplementary information; (**c**) The relative protein expression level was expressed as fold changes over that of the C57BL/6 control group. The bars show the mean ± SEM (n = 4). ^*^ *P* < 0.05 compared with the group treated with RivX alone, and ^#^ *P* < 0.05 compared with the group treated with AngII alone.

Having shown that the antagonists of AT2R and Mas attenuated AngII-enhanced anticoagulant effects of rivaroxaban, we examined whether the expression levels of AT2R and Mas had changed. As expected, the mRNA expression levels of both AT2R and Mas were increased when treated with rivaroxaban in the presence of AngII compared with those treated with rivaroxaban or AngII alone (Fig. 4a). Moreover, their protein expression levels were also up-regulated when treated with both rivaroxaban and AngII (Fig. 4b and c). Therefore, the treatment with both rivaroxaban and AngII induced the expression of AT2R and Mas at both mRNA and protein levels.

### Rivaroxaban promoted TFPI expression and activity in the presence of AngII in human umbilical vein endothelial cells (HUVECs)

**Figure 5:**
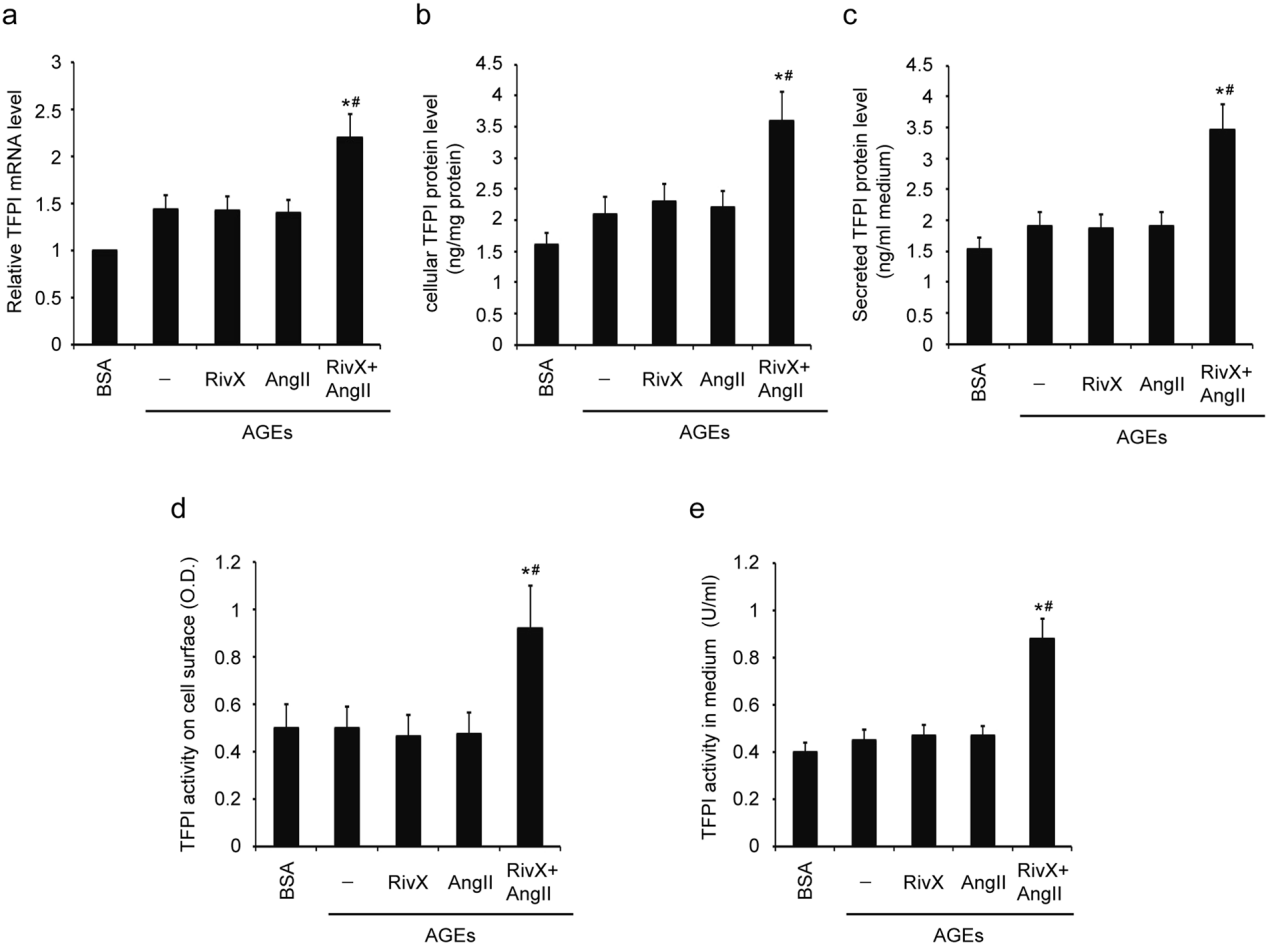
Rivaroxaban promoted TFPI expression and activity in the presence of AngII in HUVECs. HUVECs were pre-incubated with 100 μg/ml AGE-BSA and non-glycated BSA for 2 h. Cells were then treated with 30 nM rivaroxaban (RivX) or 200 nM AngII for 4 hours. (**a**) TFPI mRNA expression, (**b**) cellular TFPI protein, (**c**) TFPI secretion into the culture medium, (**d**) TFPI activity on the cell surface, and (**e**) TFPI activity in the medium were measured. The bars show the mean ± SEM (n = 4). ^*^ *P* < 0.05 compared with the parallel RivX alone group, and ^#^ *P* < 0.05 compared with the parallel AngII alone group.

To further determine whether TFPI is a crucial mediator in the increased anticoagulant effects of rivaroxaban in the presence of AngII, advanced glycation end product **(**AGE)-exposed HUVECs were subjected to a treatment with rivaroxaban plus AngII. TFPI expression and activity levels were monitored. As shown, the additional presence of AngII increased cellular TFPI’s mRNA levels (Fig. 5a), protein levels (Fig. 5b), and secretion levels (Fig. 5c) compared with the presence of rivaroxaban alone. The TFPI activities on the cell surface (Fig. 5d) and in the medium (Fig. 5e) showed the same trend as the TFPI expression. Furthermore, despite its up-regulation by AngII, the TF expression and activity displayed no significant changes between cells treated with ribaroxaban alone and co-treated with rivaroxaban and AngII (Supplementary Figure 3). In short, compared with rivaroxaban alone, the combination of rivaroxaban and AngII increased the expression and activity levels of TFPI in HUVECs.

### The AT2R and Mas antagonists abolished the up-regulated expression and activity levels of TFPI in the combination of rivaroxaban and AngII in HUVECs

**Figure 6:**
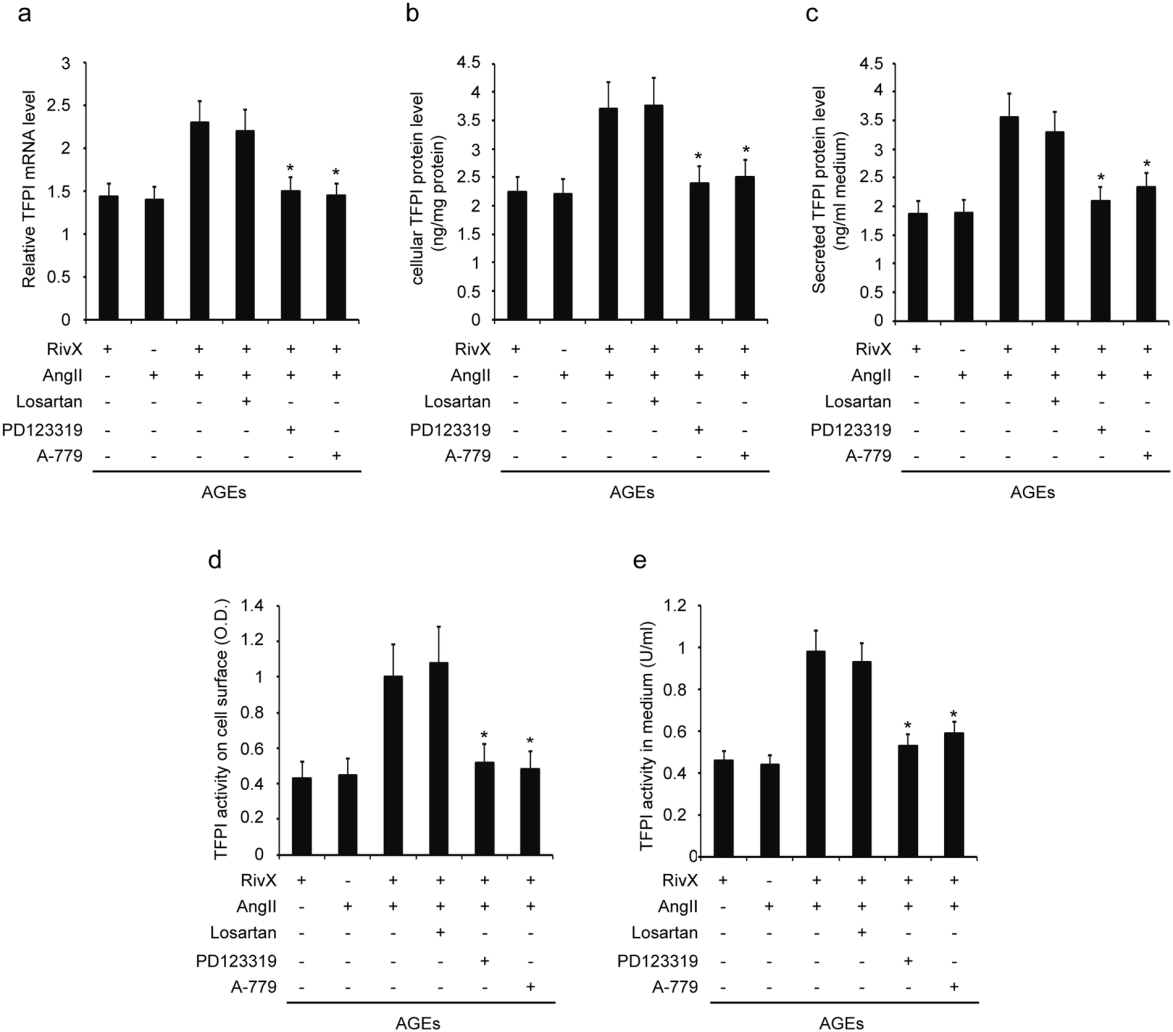
AT2R antagonist abolished rivaroxaban-promoted TFPI expression and activity in the presence of AngII. HUVECs were pre-incubated with 100 µg/ml AGE-BSA for 2 hours. Cells were then treated with 30 nM rivaroxaban (RivX) or AngII (200 nM) in the presence of AT1R antagonist Losartan (1 µM), or AT2R antagonist PD123319 (1 µM), or Mas antagonist A-779 (1 µM) for 4 h. (**a**) TFPI mRNA expression, (**b**) cellular TFPI protein, (**c**) TFPI secretion into the culture medium, (**d**) TFPI activity on the cell surface, and (**e**) TFPI activity in medium were measured. The bars show the mean ± SEM (n = 4). ^*^ *P* < 0.05 compared with the parallel group co-treated with RivX and AngII.

Subsequently, we examined whether AngII receptors were implicated in the increased TFPI expression and activity levels in HUVECs. Firstly, the combination of rivaroxaban and AngII enhanced cellular TFPI expression and secretion level. These promoted TFPI mRNA level (Fig. 6a), protein level (Fig. 6b), and secretion level (Fig. 6c) were largely abolished by PD123319 and A-779, but not by Losartan, an AT1R antagonist. Similarly, the TFPI activities on the cell surface (Fig. 6d) and in the medium (Fig. 6e) were reversed significantly by PD123319 and A-779, but not by Losartan.

For TF, its relative mRNA expression level (Supplementary Figure 4a), cellular protein level (Supplementary Figure 4b), and secreted protein level (Supplementary Figure 4c), were all down-regulated by the addition of Losartan, but were all up-regulated by the addition of PD123319 and A-779 compared with those treated with both rivaroxaban and AngII. Similarly, the TF activities on the cellular surface (Supplementary Figure 4d) and in the medium (Supplementary Figure 4e) were suppressed by the addition of Losartan, but were enhanced by the addition of D123319 and A-779 compared with those treated with both rivaroxaban and AngII. These findings suggest that the increase in TFPI expression and activity levels promoted by the combination of rivaroxaban and AngII was mediated by AT2R and Mas signaling.

### Combination of rivaroxaban and AngII did not suppress activities of MMPs in HUVECs

**Figure 7:**
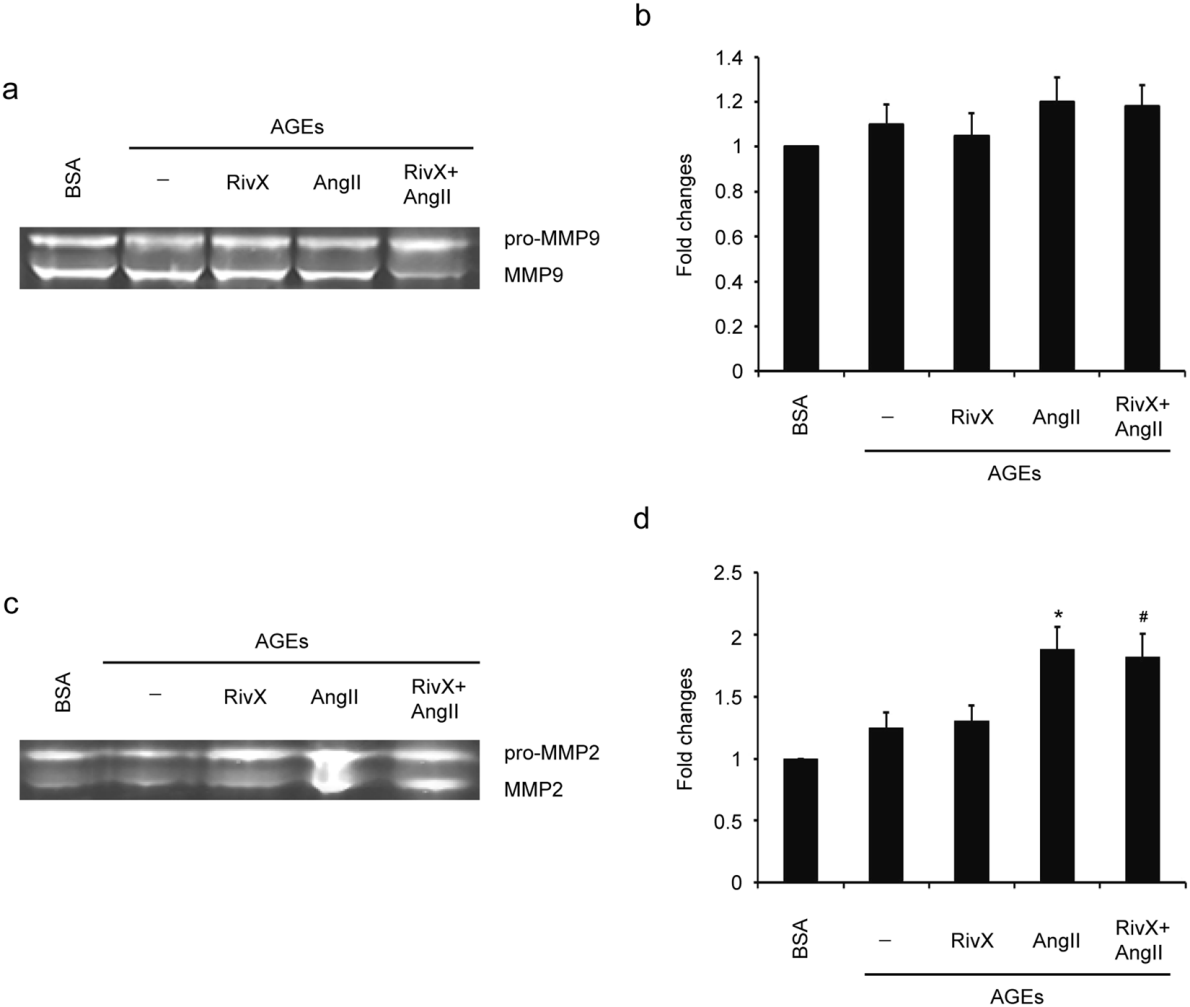
Rivaroxaban did not suppress activities of MMP9 and MMP2 in the presence of AngII in HUVECs. HUVECs were pre-incubated with 100 μg/ml AGE-BSA and non-glycated BSA for 2 h. Cells were then treated with 30 nM rivaroxaban (RivX) or 200 nM AngII for 4 hours. (**a**) MMP9 activity in the cell culture medium was determined through gelatin zymography analysis. (**b**) MMP9 activity was expressed as fold changes over that of the BSA control group. The bars show the mean ± SEM (n = 3). (**c**) MMP2 activity in the cell culture medium was determined through gelatin zymography analysis. (**d**) MMP2 activity was expressed as fold changes over that of the BSA control group. Cropped images for the gels were shown in (**a**) and (**c**). And images for the full-length gels can be found in the supplementary information. The bars show the mean ± SEM (n = 3). ^*^ *P* < 0.05 compared with the parallel blank group, and ^#^ *P* < 0.05 compared with the parallel RivX alone group.

Several metalloproteinases (MMPs), such as MMP9 and MMP2, can cleave TFPI, and thus render it considerably inactive^8^. Thus it is possible that the change in TFP1 expression level and activity might result from the change in the expression level or activity of MMPs. To test this hypothesis, we determined whether the secretion of both latent MMPs and proteolytically active MMPs were altered by a combination of rivaroxaban and AngII. AGE-exposed HUVECs were subjected to a treatment with rivaroxaban and AngII, and the MMP9 and MMP2 levels were measured through gelatin SDS-PAGE zymography. The additional presence of AngII did not suppress the activity of secreted MMP9 (Fig. 7a and b) and MMP2 (Fig. 7c and d) compared to treatment with rivaroxaban alone. Therefore, the increased TFPI expression after treatment with rivaroxaban plus AngII was less likely caused by the actions of MMPs in HUVECs.

## Discussion

In this study, we showed that AngII promoted the anticoagulant effects of rivaroxaban in KKAy mice. In particular, treatment with rivaroxaban plus AngII promoted TFPI activity *in vivo*, and induced TFPI expression and activity in AGE-exposed HUVECs. The AT2R and Mas antagonists attenuated the AngII-enhanced anticoagulant effects of rivaroxaban *in vivo*, and abolished the increased expression and activity level of TFPI in HUVECs treated with rivaroxaban plus AngII. Taken together, these data suggest that AngII promote the anticoagulant effects of rivaroxaban via AT2R and Mas signaling.

Rivaroxaban is an oral anticoagulant that directly inhibits the activity of FXa and prothrombinase complex. It has been used to prevent stroke and systemic thromboembolism in patients with non-valvular atrial fibrillation. In addition, it has also been administered to avoid venous thromboembolic events after orthopedic surgery^9^. Even though rivaroxaban is a fairly new medicine with good security, its adverse effects are still inevitable. Bleeding is one of these effects and can sometimes be life-threatening. In a clinical therapy for patients with non-valvular atrial fibrillation who received oral anticoagulant treatment, rivaroxaban increased the rates of both major and minor clinically relevant gastrointestinal bleeding compared with warfarin^3^. These results highlight the risk of hemorrhage associated with rivaroxaban and call for research into the identification of risk factors and the illustration of molecular mechanisms underlying these adverse effects.

AngII is the primary mediator of RAS. AngII-induced hypertension is associated with accelerated thrombus development in arterioles, and AngII commonly induces a hypercoagulation status^10^. In our study, we observed that the *in vivo* anticoagulant effects of rivaroxaban were promoted by AngII (Fig. 1a). As the primary function of AngII is procoagulation, this observation suggested the possible crosstalk between rivaroxaban and AngII. We set out to understand the underlying molecular mechanisms.

Several studies have indicated that AngII induces TF expression in cardiovascular systems^11–13^. However, the effects of AngII on TFPI are controversial^11, 12^. In our study, several lines of evidence suggest that TFPI might be a mediator in this anticoagulation processes. First, TFPI level and activity in the plasma were enhanced significantly after treatment with rivaroxaban plus AngII (Fig. 1e and f). Second, rivaroxaban induced both TFPI expression and activity in the presence of AngII in AGE-exposed HUVECs (Fig. 5). Unlike TFPI, despite a promotion by AngII, in contrast with the results from rivaroxaban alone group, TF expression and activity were unaffected by the combination of rivaroxaban and AngII (Fig. 1c,d, and Supplementary Figure 3). These findings suggest that a high level of AngII may increase the bleeding risk during the administration of rivaroxaban and that TFPI may be a crucial mediator.

Inflammation and coagulation interact with each other. Several pro-inflammatory cytokines, such as IL-1β, IL-6, IL-12, and TNF-α, function as procoagulant mediators^1^. However, combined administration of rivaroxaban and AngII in KKAy mice failed to suppress the plasma levels of IL-1β, IL-6, IL-12, and TNF-α (Fig. 2). Therefore, we cannot attribute pro-inflammatory cytokines to the up-regulated anticoagulant effects of rivaroxaban in the presence of AngII.

AngII can bind to at least two types of high-affinity receptors, namely, AT1R and AT2R. Currently, most of the well-known pathophysiologic effects of AngII are mediated by AT1R. By contrast, the functions of AT2R are less well understood. Several studies indicated that the expression of AT2R reoccurs in adults under pathophysiologic conditions, such as cardiovascular injury and remodeling^4, 5^. AT2R activation also counteracts most effects of AT1R by inducing cell apoptosis, reducing inflammation and oxidative stress, and promoting vasodilation^4^. Therefore, appropriate balance between AT1R and AT2R activation may be critical in maintaining body homeostasis.

In the current study, the prolonged bleeding time caused by co-administration of rivaroxaban and AngII was largely abolished by the AT2R specific antagonist. By contrast, the AT1R specific antagonist exerted no effect (Fig. 3a), thus confirming the novel anticoagulation function of AT2R, which was documented by Fang *et al.* previously^14^. Moreover, *in vivo* and *in vitro* experiments showed that in accordance with bleeding time, the up-regulated expression levels and activities of TFPI were also reversed by the AT2R specific antagonist, whereas AT1R antagonist exerted no effect (Figs 3c,d and 6). Apart from the AT2R, receptor Mas has been demonstrated to counteract actions of the AT1R. Moreover, both AT2R and Mas mediated a multitude of strikingly similar tissue protective and regenerating processes^15^. Similar to AT2R antagonist, Mas antagonist suppressed the extension of bleeding time and promotion of TFPI expression and activity significantly (Figs 3c,d and 6). However, concerning AT2R and Mas receptor, antagonists for one receptor are able to inhibit the action of agonists for the respective other receptor, indicating that there may be a functional interaction of both receptors^15^. Therefore, observations in our study did not determine that anticoagulation function of both receptors came from their interaction or individual behavior.

Previously, Fang *et al.* reported that Bdkrb2-deleted mice had increased bleeding time which was due to promoted expression levels of AT2R and Mas receptor^14^. Therefore, we also determined the expression levels of AT2R and Mas in KKAy mice. However, KKAy mice showed no altered expression levels of both receptors (Fig. 4). Moreover, treatment with rivaroxaban alone or AngII alone did not change their expression levels as well (Fig. 4). Interestingly, combination of rivaroxaban and AngII induced both AT2R and Mas expression levels (Fig. 4), leading to a hypocoagulation status. Concerning these observations, there is a question how the expression levels of AT2R and Mas are induced under the condition of rivaroxaban plus AngII. Could the coagulantion system and the RAS crosstalk with each other to impact their biological balance or body homeostasis? This problem need deep studies. Overall, AngII-enhanced anticoagulant action of rivaroxaban was likely mediated by AT2R and Mas.

With regard to the procoagulant factor TF, we obtained complicated results. Firstly, the combination of rivaroxaban and AngII did not suppress TF expression and activity (Fig. 1c,d, and Supplementary Figure 3). This observation did not coincide with the results of bleeding time. Secondly, the AT1R antagonist suppressed TF expression and activity in the context of a treatment with rivaroxaban plus AngII (Supplementary Figures 2 and 4), a result that coincide with previous reports that AngII induces TF expression through the AT1R^16, 17^. Finally, the AT2R and Mas antagonists promoted TF expression and activity under the same condition (Supplementary Figures 2 and 4). A possible explanation is that the inhibition of TFPI by the AT2R or Mas blocker increased TF synthesis and activity. Overall, it was unlikely that the decreased TF level was responsible for a hypocoagulation status induced by the combination of rivaroxaban and AngII. The alterations of TF induced by AT2R and Mas antagonists were largely due to TFPI actions. A model was proposed based on these findings (Supplementary Figure 5). In the model, rivaroxaban alone has little effect on the expression and/or activity of TFPI or TF. Similarly, AngII alone does not affect TFPI. However, it promotes the expression and/or activity of TF via AT1R. Lastly, in AT2R/Mas dependent manner, rivaroxaban plus AngII induces TFPI, which then suppresses and counteracts the expression and/or activity of TF enhanced by AngII.

The release of MMPs from inflammatory cells may promote thrombosis by cleaving the TFPI protein and suppressing its activity. However, treatment with rivaroxaban plus AngII did not suppress the activity of secreted MMP9 and MMP2 in HUVECs (Fig. 7). Therefore, the increased TFPI expression level is less likely caused by suppressed activation of MMP9 and MMP2 in the context of treatment with rivaroxaban plus AngII. Additional studies are required to identify novel mediators implicated in increased TFPI expression and activity by AT2R and Mas signaling after treatment with rivaroxaban plus AngII. However, there is some limitation using HUVECs as the cell model. Endothelial cells show multiple phenotypes and functions depending on their origin of the blood vessels. For example, significant differences in phenotypes and functions have been observed between HUVECs and aortic endothelial cells. Therefore, the results obtained in HUVECs need to be verified in other types of endothelial cells.

In summary, our results, for the first time, demonstrate that AngII promotes the anticoagulant effects of rivaroxaban via AT2R and Mas signaling in an experimental mouse model with type 2 diabetes mellitus, and TFPI is likely a key mediator. The findings of this study are highly significant for the clinical administration of rivaroxaban. However, the deleterious effects of rivaroxaban in the presence of AngII was only studied for a particular dose. Additional studies are needed to further characterize the effects of their combination at various doses, in order to guide the precise usage of rivaroxaban in the presence of AngII in clinical and pharmacological applications.

## Methods

### Animal experiments

Seven-week-old male KKAy mice were purchased from HFK Bioscience (Beijing, China). All experimental procedures conducted in this study were performed in accordance with the relevant guidelines and regulations approved by the Institutional Animal Care and Use Committee at Peking Union Medical College. A total of 30 mice were provided with water and chow diet and randomly divided into three groups (10 mice per group). The first group was fed with chow diet alone. The mice in the second group received chow diet and were administered with 5 mg rivaroxaban/kg body weight/day for 15 d via a gastric feeding tube. The third group was administered with AngII (1,500 ng/kg/min; Sigma, USA) in addition to rivaroxaban for 2 weeks starting on the first day after rivaroxaban administration. For oral administration, micronized rivaroxaban powder was dissolved in 100% DMSO (20 mg/56 ml) and diluted with demineralized water to the final concentration of DMSO ≤ 1.0%. For osmotic pump preparation, AngII was dissolved in saline. AngII and saline were infused by using an Alzet model pump (1004).

At the same time of rivaroxaban and AngII administration, the AT1R antagonist olmesartan medoxomil (Daiichi Sankyo, Japan) dissolved in 0.5% carboxymethyl cellulose was administered orally at a dose of 0.5 mg/kg/day for 2 weeks. The AT2R antagonist PD123319 (3 mg/kg/day; Sigma, USA) and Mas antagonist A-779 (2 mg/kg/day; LKT Labs, USA), dissolved in a saline solution, were co-infused into mice with AngII for 2 weeks.

### Assessment of coagulation effect *in vivo*

Tail bleeding times in mice were determined. Briefly, 1 mm distance from the tail tip was amputated and immersed in 0.9% isotonic saline at 37 °C. The time to complete arrest of bleeding (no blood flow for 1 min) was determined. Furthermore, mice were anesthetized with isoflurane gas, and blood was collected with a cardiac puncture. Plasma thrombin-antithrombin (TAT) complexes were assayed through AssayMax mouse ELISA (AssayPro, St. Charles, MO). Plasma TF and TFPI levels were determined with Imubind^TM^ total TF and TFPI ELISA kit, respectively. Soluble thrombomodulin (sTM) was quantified by ELISA (Stratech Scientific Ltd.). Plasma von Willebrand factor (vWF) was detected with vWF ELISA kit (Ramco Laboratories Inc., Stafford, TX, USA). Plasma TF and TFPI activities were determined with the Actichrome^TM^ TF and TFPI activity assay kits, respectively.

### ELISA analysis of proinflammatory cytokines

Necropsy blood samples were collected from mice via a cardiac puncture. The plasma concentrations of IL-1β, IL-6, IL-12, and TNF-α were determined with corresponding ELISA kits (R&D Systems) in accordance with the manufacturer’s instructions.

### Western blot analysis

The mouse aortae were harvested and Western blot analysis was performed according to protocols described previously^18^. Antibodies to AT2R and Mas were purchased from Santa Cruz Biochemicals and Nova Biologicals LLC, respectively. Antibody to GAPDH was from Cell Signaling Technoligy. To quantify protein levels, we subtracted background and normalized the value to that of β-actin. The expression level for each group was then expressed as fold change over that of the control group.

### Preparation of AGEs

AGEs were prepared as described previously^19^. Briefly, BSA (25 mg/ml) was incubated under sterile conditions with 0.1 M D-glyceraldehyde in 0.2 M phosphate buffer (pH 7.4) for 7 d. After incubation, the AGEs were dialyzed against phosphate buffered saline to remove unbound sugars. Non-glycated BSA control was incubated under the same conditions except for the absence of reducing sugars. The protein levels of AGE were measured through a bicinchoninic acid (BCA) assay.

### Cell culture and treatment

HUVECs (Sciencell, USA) were cultured in an endothelial cell medium (ECM) supplemented with endothelial cell growth supplement (ECGS), 5% fetal bovine serum (FBS), and a penicillin/streptomycin (P/S) solution (Sciencell, USA) in an incubator at 37 °C with 5% CO_2_. Cells of passage no. 4–6 were grown until confluence and used for the experiments. Rivaroxaban, AGE, AngII, AT1R antagonist, AT2R antagonist, and Mas antagonist were supplemented in a medium lacking FBS and ECGS.

### Reverse transcription quantitative real-time PCR (RT-qPCR) analysis

Total RNA was isolated from aortae and HUVECs by using a Trizol reagent (Invitrogen). The qPCR was conducted with a Bio-RAD iQ5 Multicolor Real-Time PCR Detection System with SYBR Green as the fluorescent dye and ROX (Takara) as the reference dye. The sequence of the specific primers used were as follows: mouse AT2R forward 5′-GGTCTGCTGGGATTG CCTTAATG-3′, reverse 5′-ACTTGGTCACGGGTAATTCTGTTC-3′; mouse Mas forward 5′-TCTACTTGGGGATCGACTGG-3′, reverse 5′-GCACTGCTGTTGATGCAGAT-3′; mouse GAPDH forward 5′-ACTCCACTCACGGCAAATTC-3′, reverse 5′-TCTCCATGGTGG TGAAGACA-3′; human TFPI forward 5′-ACTCGACAGTGCGAAGAA-3′, reverse 5′-GGCATCCACCATACTTGA-3′; human GAPDH forward 5′-GACCCCTTCATTGACCTC-3′, reverse 5′-GCTAAGCAGTTGGTGGTG-3′. Relative quantification was performed through ΔΔCt method with GAPDH as the endogenous control. Each sample was run at least in triplicate, and the mRNA expression levels were expressed as fold change over that of the control.

### Measurement of cellular and secreted protein levels of TFPI

HUVECs were cultured on 24-well plates. The culture supernatants were collected and the HUVECs were lysed in 100 μL extraction buffer (50 mmol/L Tris, 100 mmo/L NaCl, 0.1% (w/vol) Triton X-100, pH 7.4) and centrifuged at 20,000 × g to clear the debris prior to analysis. TFPI levels were then determined with Imubind^TM^ TFPI ELISA (American Diagnostica) following the manufacturer’s protocol, and expressed as ng/mg protein.

### Measurement of cellular TFPI activity

The TFPI activity in the conditioned culture medium was determined with Actichrome^TM^ TFPI activity assay kits (American Diagnostica) in accordance with the manufacturer’s protocol. TFPI activity was calculated according to the corresponding standards provided by the manufacturer and expressed as U/ml. One unit of TFPI activity refers to the activity of TFPI present in 1 ml normal human serum (as defined by the manufacturer). To measure the cell-surface TFPI activity, confluent monolayers of HUVECs on 96-well microplates were washed twice with PBS, and reagents were added directly to the microplate wells. TFPI activity was expressed as optical density, following correction with values for chromogen added to wells without cells.

### Gelatin zymography analysis

The cell culture medium was harvested and the MMP9 activity was determined through gelatin zymography. The zymogram gels consisted of 10% polyacrylamide gel incorporated with 0.1% gelatin. After electrophoresis, the gels were washed in 2.5% Triton X-100 for 1 h, incubated with collagenase buffer at 37 °C for 18 h, stained for 1 h with 0.1% Coomassie brilliant blue, and destained with water. Gelatinolytic activities were visualized as a clear zone, and spot density was measured with a digital imaging analysis system (Alpha Innotech, Mt. Prospect, IL). The expression levels are expressed as fold changes over that of the control.

### Statistical analysis

All values are presented as the mean ± standard error of the mean (SEM). The statistical significance of differences was evaluated by student’s *t*-test or Analysis of Variance using SPSS10 software.

## Electronic supplementary material

Supplementary information
