# Supplementary material for: Angiotensin II promotes the anticoagulant effects of rivaroxaban via angiotensin type 2 receptor signaling in mice
Source: Sci Rep. 2017 Mar 23;7:369. doi: 10.1038/s41598-017-00473-5 (PMC5428434; doi:10.1038/s41598-017-00473-5)

SUPPLEMENTARY INFORMATION

Angiotensin II promotes the anticoagulant effects of rivaroxaban via angiotensin type 2 receptor signaling in mice

Dan Yang[a], Junjie Shao[a], Ruifeng Hu[a], Haimei Chen[a], Ping Xie[a,b,*], Chang Liu[a*]

[a] Key Laboratory of Bioactive Substances and Resources Utilization of Chinese Herbal Medicine from Ministry of Education, Institute of Medicinal Plant Development, Chinese Academy of Medical Sciences and Peking Union Medical College, Beijing 100193, PR China.

[b] Department of Pathology, Shanghai KingMed Diagnostics, Shanghai 201321, PR China.

* Corresponding author. Tel.: +086 010-57833111; Fax: +086 010-62899715. E-mail address: cliu6688@yahoo.com (Chang Liu)

xieping76@hotmail.com (Ping Xie)

1. Supplementary Figures

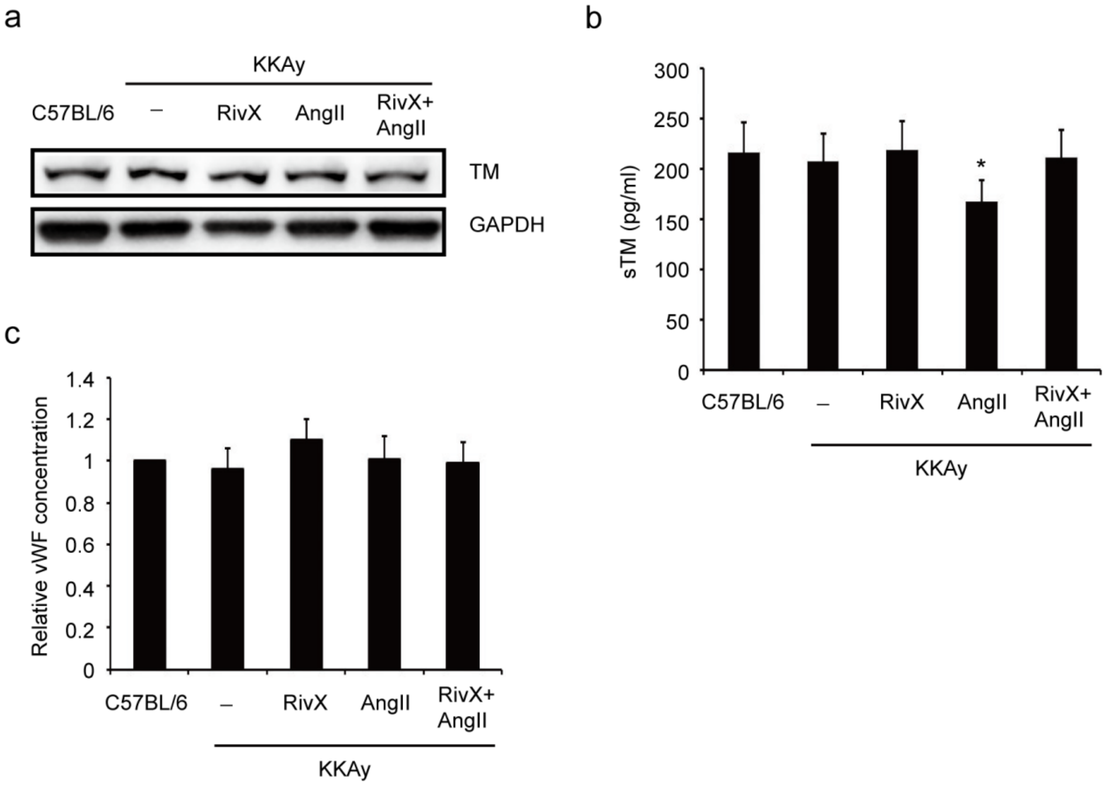

Supplementary Figure 1

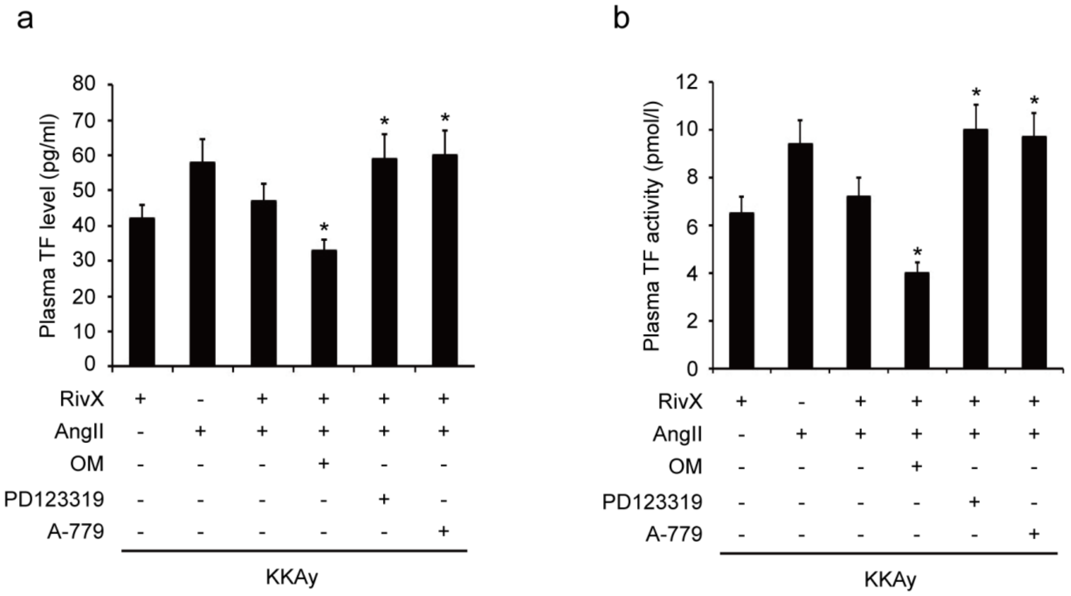

Supplementary Figure 2

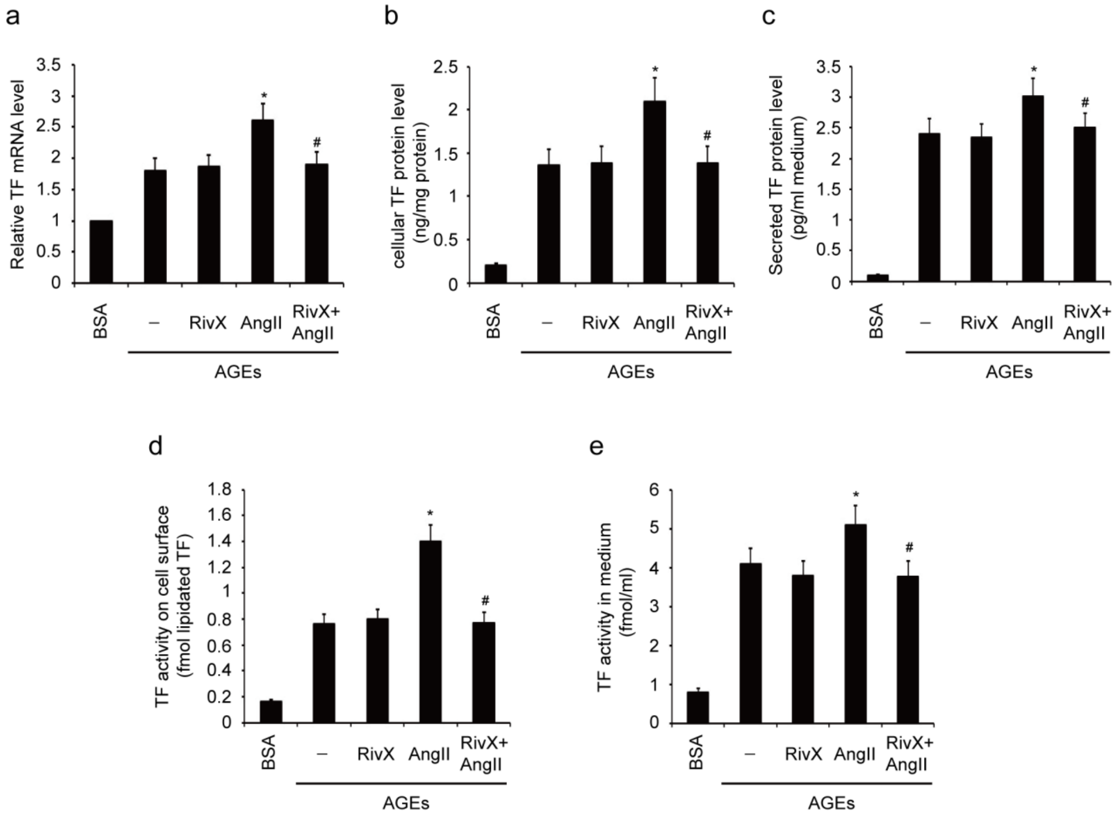

Supplementary Figure 3

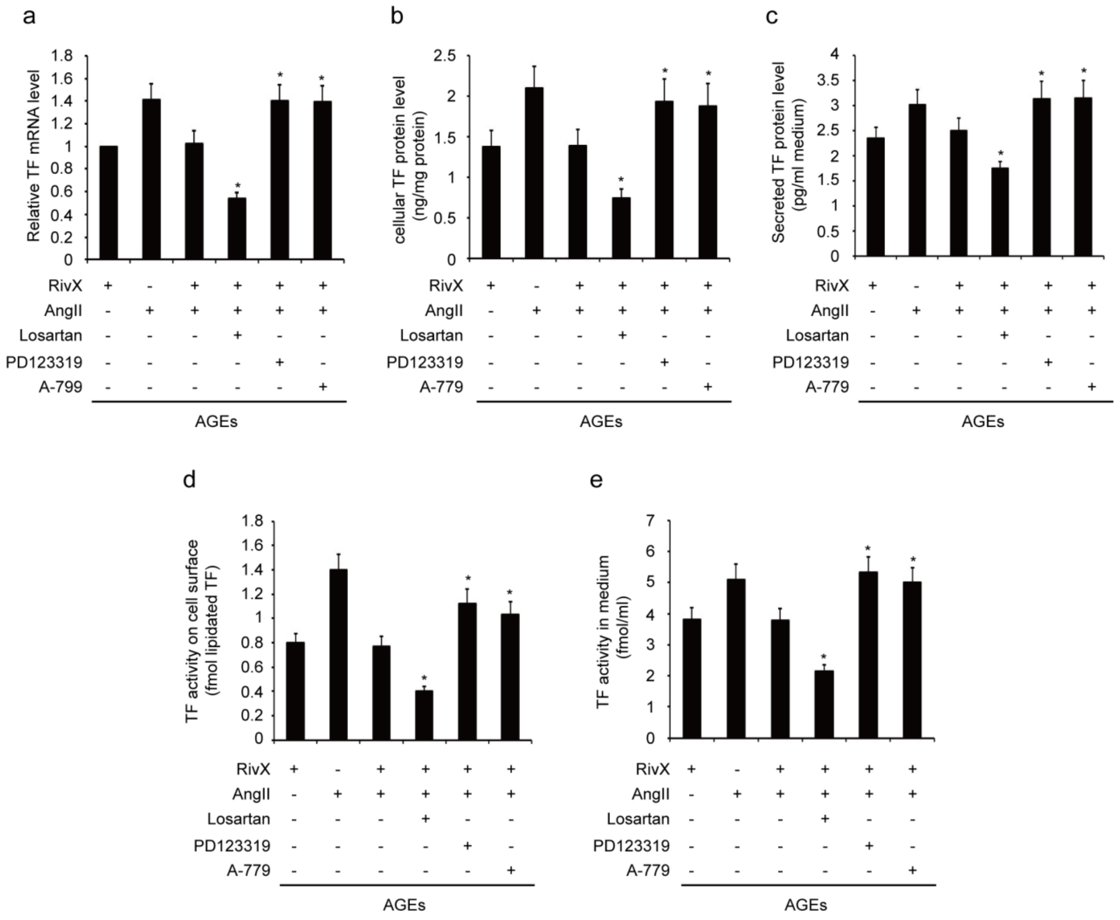

Supplementary Figure 4

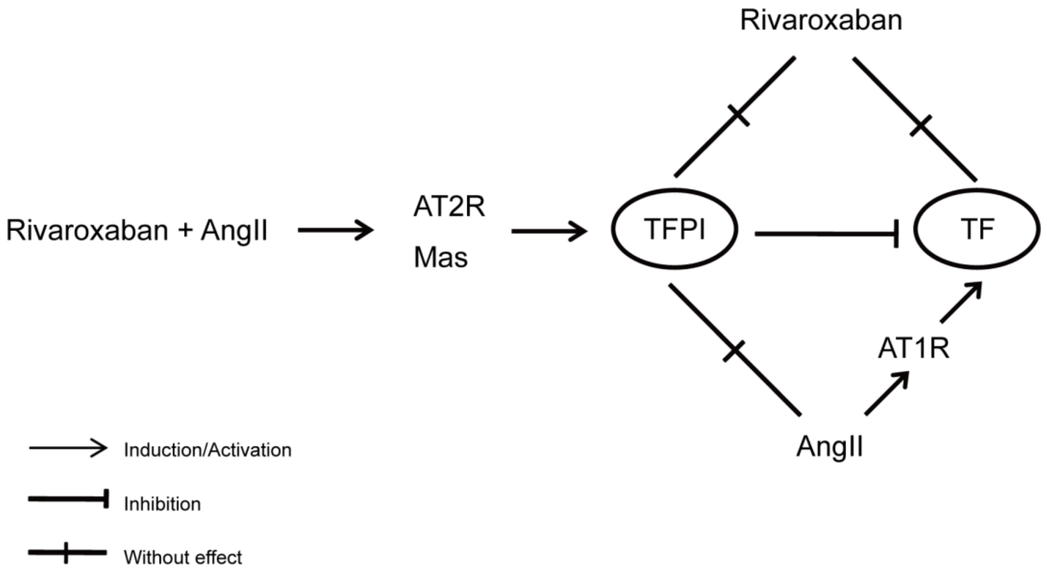

Supplementary Figure 5

Supplementary Figure legends

Supplementary Figure 1. Combination of rivaroxaban and AngII did not affect the levels of thrombomodulin and von Willebrand factor. Mice were administered orally with rivaroxaban (RivX, 5 mg/kg body weight/day), or infused with AngII (1,500 ng/kg/min), or co-treated with RivX and AngII for 2 weeks. (a) Under isoflurane anesthesis, the aortae were harvested. The protein expression levels of thrombomodulin (TM) and GAPDH were detected using Western blot analysis. Representative images were shown (n=3). Blood was collected by cardiac puncture to determine the plasma levels of soluble thrombomodulin (sTM) (b) and von Willebrand factor (vWF) (c) using ELISA. The bars show the mean ± SEM (n=5). $^{*}P<0.05$, compared with the KKAy blank group.

Supplementary Figure 2. AngII receptor antagonists affected plasma TF levels and activities. Mice were administered with rivaroxaban (RivX, 5 mg/kg body weight/day) or AngII (1,500 ng/kg/min) in the presence of AT1R antagonist olmesartan medoxomil (OM, 0.5 mg/kg/day), or AT2R antagonist PD123319 (3 mg/kg/day), or Mas antagonist A-779 (2 mg/kg/day) for 2 weeks. Blood was collected by cardiac puncture to determine the plasma levels (a) and activities (b) of tissue factor (TF). The bars show the mean ± SEM (n=4). $^{*}P<0.05$, compared with the group co-treated with RivX and AngII.

Supplementary Figure 3. Rivaroxaban showed no effect on TF expression and activity in the presence of AngII in the endothelial cells. HUVECs were pre-incubated with 100 μg/ml AGE-BSA and non-glycated BSA for 2 h, and then cells were treated with 30 nM rivaroxaban (RivX) or 200 nM AngII for 4 h. (a) The TF mRNA expression, (b) cellular TF protein, (c) TF secretion into culture medium, (d) TF activity on cell surface, and (E) TF activity in medium were measured. The bars show the mean ± SEM (n=3). $^{*}P<0.05$, compared with the parallel AGEs control group; $^{\#}P<0.05$, compared with the AngII group,

Supplementary Figure 4. AngII receptor antagonists affected endothelial TF levels and activities. HUVECs were pre-incubated with 100 μg/ml AGE-BSA for 2 h, and then cells were treated with 30 nM rivaroxaban (RivX) or AngII (200 nM), in the presence of AT1 antagonist losartan (1 μM), or AT2 antagonist PD123319 (1 μM), or Mas antagonist A-779 (1 μM) for 4 h. (a) The TF mRNA expression, (b) cellular TF protein, (c) TF secretion into culture medium, (d) TF activity on cell surface, and (e) TF activity in medium were measured. The bars show the mean ± SEM (n=3). $^{*}P<0.05$, compared with the group co-treated with RivX and AngII.

Supplementary Figure 5. A proposed model explaining the effects of rivaroxaban and AngII on the expression and/or activity of TFPI and TF.

2. Full-length blots/gels for those shown in the main text

2.1. Full-length blot for AT2R in the Figure 4b.

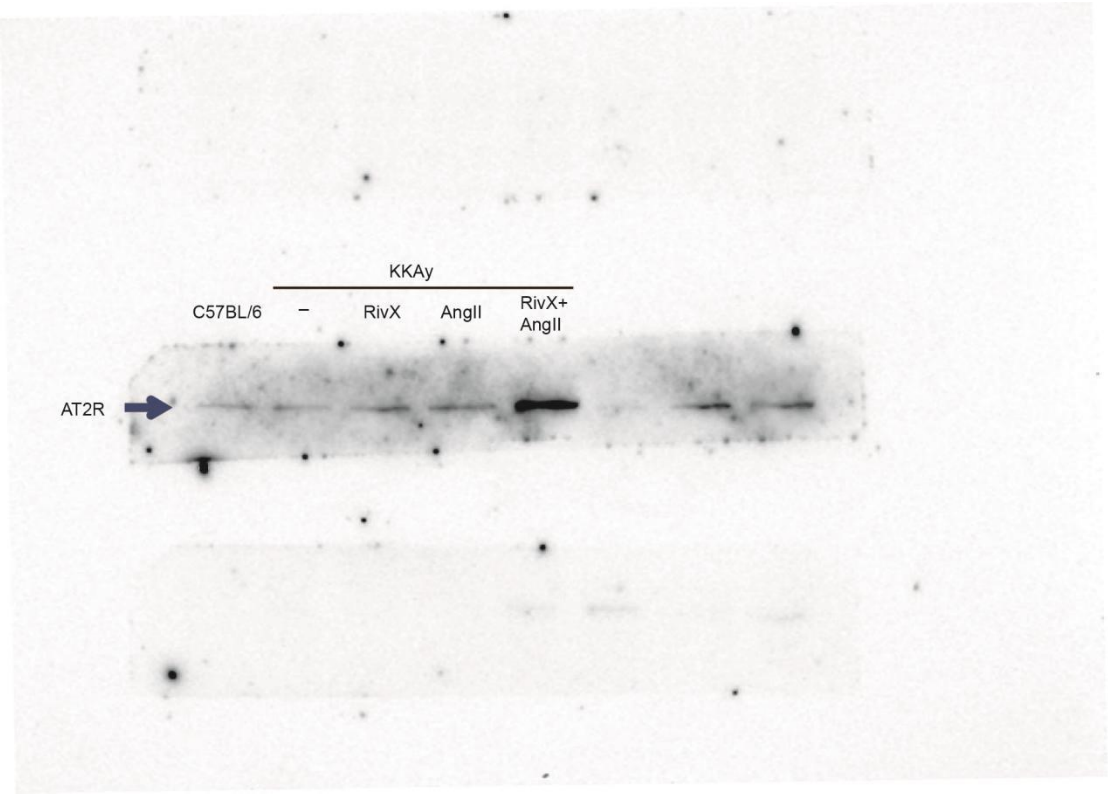

2.2. Full-length blot for Mas in the Figure 4b.

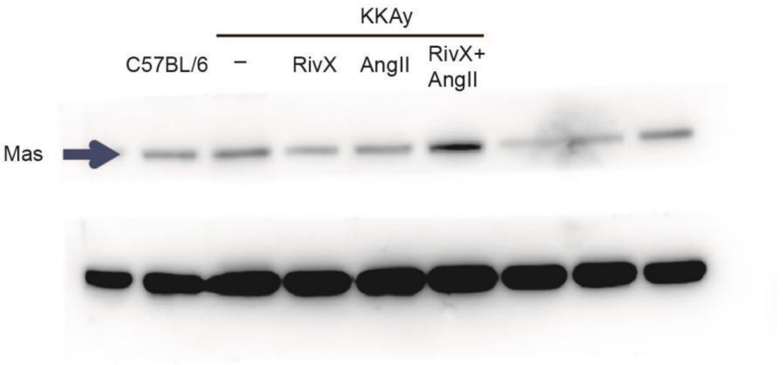

2.3. Full-length blot for GAPDH in the Figure 4b.

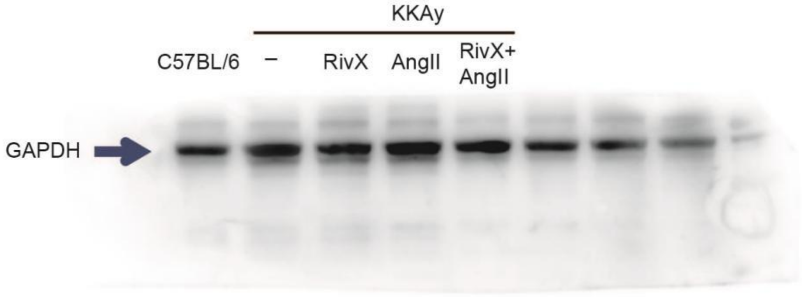

2.4. Full-length gel for MMPs in the Figure 7a and c.

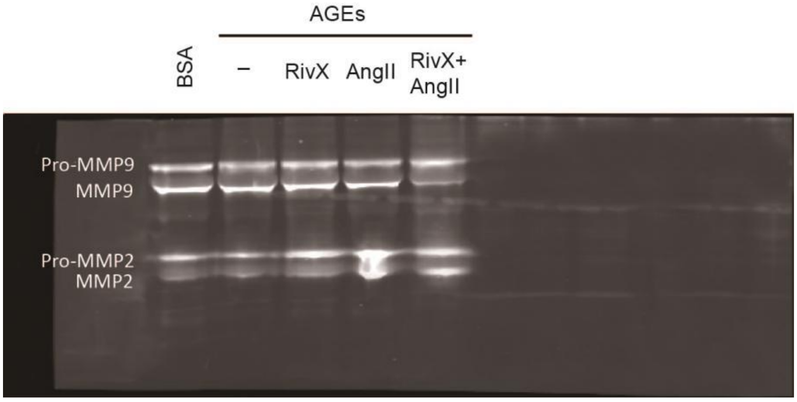

2.5 Full-length blot for TM in the Supplementary Figure 1a.

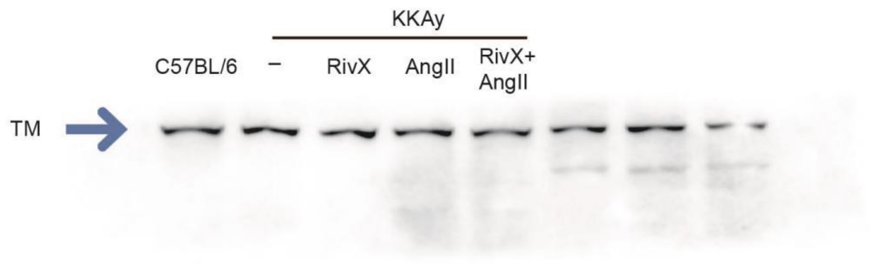

2.6. Full-length blot for GAPDH in the Supplementary Figure 1a.

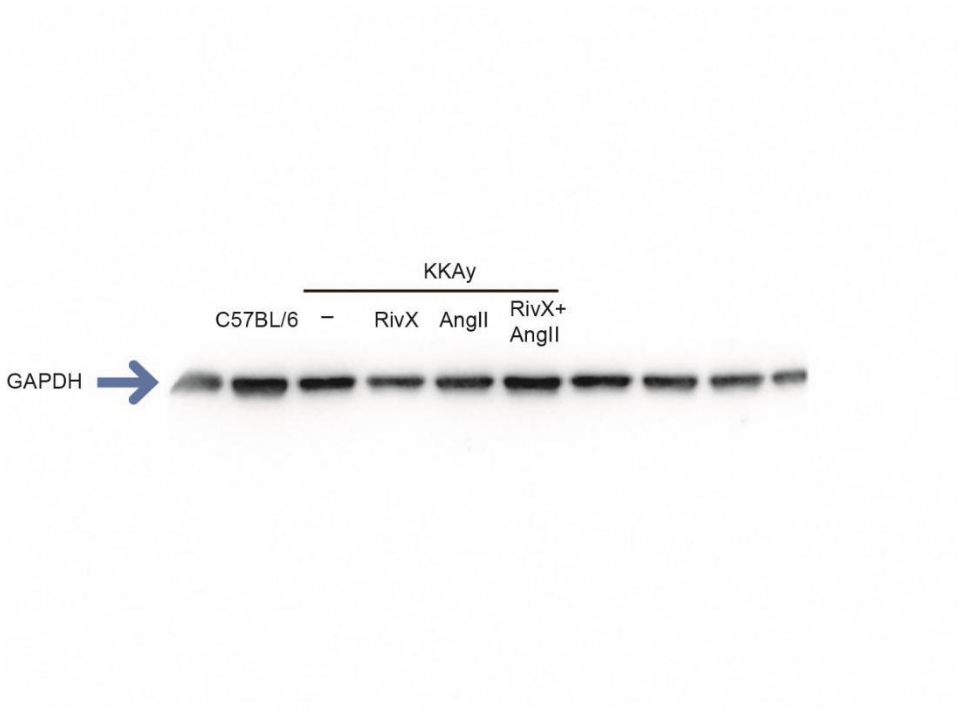

Supplement: Supplementary file 1 — Supplementary information [file 41598_2017_473_MOESM1_ESM.pdf]
